# Prevention and contrast of child abuse and neglect in the practice of European paediatricians: a multi-national pilot study

**DOI:** 10.1186/s13052-021-01055-y

**Published:** 2021-05-03

**Authors:** Paola Nigri, Giovanni Corsello, Luigi Nigri, Donjeta Bali, Giorgina Kuli-Lito, Doina Plesca, Tudor Lucian Pop, Angel Carrasco-Sanz, Leyla Namazova-Baranova, Julije Mestrovic, Mehmet Vural, Ida Giardino, Laszlo Losonczi, Eli Somekh, Maria Teresa Balducci, Massimo Pettoello-Mantovani, Pietro Ferrara

**Affiliations:** 1 Campus Bio-Medico University Medical School, Rome, Italy; 2 Italian Society of Pediatrics, Rome, Italy; 3 Institute of Pediatrics, University of Palermo, Palermo, Italy; 4 Italian Federation of Pediatricians, Rome, Italy; 5 Albanian Pediatric Society, Tirana, Albania; 6 Romanian Society of Paediatrics, Bucharest, Romania; 7 Romanian Society of Social Pediatrics, Cluj, Romania; 8 European Confederation of Primary Care Pediatricians, Lyon, France; 9 Russian Academy of Pediatrics, Moscow, Russia; 10 Croatian Society of Pediatrics, Zagreb, Croatia; 11 Turkish Pediatric Association, Istanbul, Turkey; 12 Department of Clinical and Experimental Medicine, University of Foggia, Foggia, Italy; 13 Association of Hungarian Primary Care Paediatrician, Budapest, Hungary; 14 Department of Pediatrics, Mayanei Hayeshuah Medical Center, Bnei Brak, Israel; 15 Local Health District, Poly-ambulatory, Statistic service, ASL10, Bari, Italy; 16 European Paediatric Association, Union of National European Pediatric Societies and Associations, Berlin, Germany

**Keywords:** Maltreatment, Child, Violence, Abuse, Neglect

## Abstract

**Background:**

Child abuse and neglect, or maltreatment, is a serious public health problem, which may cause long-term effects on children’s health and wellbeing and expose them to further adulthood vulnerabilities. Studies on child maltreatment performed in Europe are scarce, and the number of participants enrolled relatively small. The aim of this multi-national European pilot study, was to evaluate the level of understanding and perception of the concepts of child abuse and neglect by European paediatricians working in different medical settings, and the attitude toward these forms of maltreatment in their practice.

**Methods:**

The study was performed by a cross-sectional, descriptive, online survey, made available online to European paediatricians members of 50 national paediatric, who belonged to four different medical settings: hospital, family care, university centres and private practice.

The questionnaire, designed as a multiple choice questions survey, with a single answer option consisted of 22 questions/statements. Frequency analyses were applied. Most of the data were described using univariate analysis and Chi-squared tests were used to compare the respondents and answers and a significance level of *p* ≤ 0.05 applied.

**Results:**

Findings show that European paediatricians consider the training on child maltreatment currently provided by medical school curricula and paediatric residency courses to be largely insufficient and continuing education courses were considered of great importance to cover educational gaps. Physical violence was recognized by paediatricians mostly during occasional visits with a significant correlation between detecting abuse during an occasional visit and being a primary care paediatrician. Results also showed a reluctance by paediatricians to report cases of maltreatment to the competent judicial authorities.

**Conclusions:**

Data of this study may provide useful contribution to the current limited knowledge about the familiarity of European paediatricians with child maltreatment and their skills to recognize, manage and contrast abusive childhood experiences in their practice. Finally, they could provide local legislators and health authorities with information useful to further improve public health approaches and rules able to effectively address shared risk and protective factors, which could prevent child abuse and neglect from ever occurring.

## Background

Child abuse and neglect (CAN), or maltreatment [1], is a serious public health problem, which may cause long-term effects on children’s health and wellbeing and expose them to further adulthood vulnerabilities [2, 3]. According to recent reports, in the United States about 1 in 7 children experience CAN each year and in 2018 nearly 1770 children died of abuse and neglect ^4.^ In Europe, the 2018 World Health Organization status report on child maltreatment prevention, estimates that this phenomenon involves at least 55 million children living in the Region [5]. Poor socio-economic conditions are recognized to be important causative factors [6]. Children living in poverty are particularly exposed to abuse and neglect, and the rates of these type of abusive experiences are 5 times higher for children living in families with low socio-economic status compared to children from families with higher socio-economic status [7]. Child maltreatment is also costly [3]. In 2015, the total lifetime economic burden associated with child abuse and neglect the United States was approximately $428 billion, causing an economic burden comparable to the cost of other high profile public health problems, such as type 2 diabetes and stroke [4, 6].

Child abuse and neglect are part of the adverse childhood experiences (ACE’s) suffered by individuals under the age of 18 and usually caused by a parent, caregiver, or different person in a custodial role, which results in harm, potential for harm, or threat of harm to a child [8, 9]. In general, abuse refers to usually deliberate acts of commission, while neglect refers to acts of omission [10, 11]. The most common forms of maltreatment (Table 1) are related to other forms of violence through shared risk and protective factors [12]. Therefore, prevention of child abuse and neglect greatly contributes to prevent other forms of violence. However, preventive programs addressing ACE’s are largely based on the knowledge and capability to recognize abusive childhood experiences by healthcare professionals, particularly paediatricians [13], whose ability to recognize these events needs to be regularly updated and implemented [2].

**Table 1:** Common forms of Adverse Childhood Experiences (ACE) in minors

MOST COMMON FORMS OF CHILD MALTREATMENT	
**Physical Abuse** Legal definitions vary from country to country. However, physical abuse is broadly defined as any non-accidental physical act inflicted upon a child by a parent, caregiver, or other person who has responsibility for the child, which can result in physical injury. Examples include hitting, kicking, shaking, burning, or other shows of force against a child. **Sexual Abuse** Sexual abuse occurs when an adult or another child asks or pressures, or force a child for sexual contact. The abuser may use physical abuse, bribery, threats, tricks, or take advantage of the child’s limited knowledge of sexual matters. Most cases are perpetrated by a person familiar to the child. Sexual abuse can also include taking photos of the child, or showing them pornography through pictures, magazines, movies, online. **Emotional Abuse** Emotional abuse refers to behaviors that harm a child’s self-worth or emotional well-being. It is characterized by inattention to a child’s emotional needs, failure to provide psychological care, permitting a child to use alcohol or other drugs. In addition, children who witness domestic violence or who live with a sex offender in their homes can fall under the umbrella of emotional abuse. Examples include name calling, shaming, rejection, withholding love, and threatening **Neglect** The failure of a parent, guardian, or other caregiver to provide for a child’s basic physical and emotional needs. These needs include housing, food, clothing, education, and access to medical care. It can be in the form of physical, medical, education and emotional neglect. **Child Trafficking / Commercial Sexual Exploitation of Children (CSEC)** A commercially sexually exploited child is one under the age of 18 who engages, agrees to engage in, or offers to engage in sexual conduct in exchange for money, clothing, food, shelter, education, goods or care. Exploited youth are not “child prostitutes,” they are child victims. **Abusive Head Trauma** Infants, babies or small children who suffer injuries or death from severe shaking, jerking, pushing or pulling may have been victims of Abusive Head Trauma (AHT), formerly Shaken Baby Syndrome. The act of shaking a baby is considered physical abuse, as spinal, head and neck injuries often result from violently shaking young children. **Institutional Abuse or Neglect** Abuse or neglect which occurs in any facility for children, including, but not limited to, group homes, residential or public or private schools, hospitals, detention and treatment facilities, family foster care homes, group day care centers and family day care homes.	

Recent studies have explored child abuse and neglect in various contexts, particularly families and professionals practicing different types of job, including police officers, lawyers and teachers [14–16]. However, limited data are available about the experience of paediatricians regarding child maltreatment in their practice [2, 3]. The aim of this pilot study, promoted by the Italian Federation of Paediatricians in collaboration with the European Working Group on social paediatrics, was to evaluate both the level of understanding and perception of the concepts of child abuse and neglect by European paediatricians working in different medical settings, and the attitude toward these forms of maltreatment in their practice.

## Methods

This study, performed during February–May 2020, was planned by the Italian Federation of Paediatricians (Federazione Italiana Medici Pediatri, FIMP) in collaboration with the working group on social paediatrics of the European Paediatric Association, the Union of National European Paediatric Societies and Associations/ (EPA/UNEPSA) and the European Confederation of Primary Care Paediatricians (ECPCP). A questionnaire focusing on the knowledge, understanding, and attitude towards child abuse and neglect in their practice was made available online to European paediatricians members of the 50 national societies affiliated to EPA-UNEPSA and ECPCP, who were informed on the aims of the study and requested to voluntarily participate in the survey. The participants solicited belonged to four different medical settings: hospital (secondary and tertiary care), family care (community and primary care), university centres and private practice.

### Design of the questionnaire

A cross-sectional, descriptive, online survey, modelled on the ISPCAN Child Abuse Screening Tool Children’s Version (ICAST-C) [17], was developed in 2019 using web-based standard guidelines [18] and validated by the department of information technology of EPA-UNEPSA, in Berlin, Germany. The principles of iCAST – Intelligence Led Cyber Security Testing, introduced by the Hong Kong Monetary Authority (HKMA) in response to the ever changing cyber security landscape, were applied to its development [19]. The questionnaire was hosted on the Survio international platform [20]. The questionnaire consisted of 22 questions/statements divided into four sections [21]: *Demographic data (n.4), Awareness and attitude of about CAN (n.7), Education and competence about CAN (n.5), Practice and formal procedures about CAN (n.6).* The questionnaire was designed as a multiple choice questions survey, with a single answer option. In accordance to standard guidelines indicators of response quality in web surveys, the average time needed to fill out the questionnaire was 10 min [22].

### Ethics

The study was performed in accordance with the Declaration of Helsinki’s principles and ethical approval was received by the Ethics Committee of the European Paediatric Association/Union of National European Paediatric Societies (EC.UNEPSA.002A,12/12/2019). The study was anonymous, voluntary, with no personal or identifiable data being collected. All respondents approved their participation by informed consent and had access to the pretested forms for their final validation.

### Statistics

Frequency analyses were applied to check for data errors, and any values outside of this range were easily identified and recoded to fit into existing categories [23]. Most of the data were described using univariate analysis. Chi-squared tests were used to compare the respondents and answers and a significance level of *p* ≤ 0.05 applied. SPSS software (version 23. 2015, IBM, USA) was used for statistical analysis. The statistical analysis was elaborated by the statistical analysis unit of the European Association of Paediatrics in Berlin, Germany and further validated by the statistic unit of the Pediatric research center of the University of Foggia in collaboration with the statistical unit of the local health district of Bari, Italy.

## Results

A total of 1083 e-forms were collected. Participants (respondents) belonged to 22 European countries (Albania, Armenia, Austria, Belgium, Bosnia and Herzegovina, Croatia, France, Germany, Hungary, Ireland, Israel, Italy, Kazakhstan, Lithuania, Poland, Portugal, Romania, Russia, Slovakia, Spain, Turkey, and Slovenia), thus providing a balanced geographic distribution throughout the continent, also representative of different socio-economic local realities [24, 25]. Of those who declared their gender, more women (*n* = 716, 66.1%) than men (*n* = 368, 33.98%) contributed to the study. Greater group of respondents belong to age group 51–60 (31,3%), followed by age groups > 61 (25,7%), 41–50 (21,9%) and < 40 (21.1%). The majority of respondents were family care paediatricians working in community or primary care settings (51,6%), while hospital paediatricians working in secondary and tertiary care were 34,3%, paediatricians working in university centres 10,1% and private care paediatricians 4,0%.

### Awareness and attitude about CAN

Most of the times (49,4%), child abuse, neglect and in general violence against children, were identified by paediatricians as occasional finding during routine checks and in about one out of four cases (23,1%) during visits directly related to the matter. In 17,4% of the cases maltreatments were unreported and could be identified during emergency visits, while 10,1% of paediatricians recognized cases of child abuse and neglect in different circumstances.

**Fig. 1:**
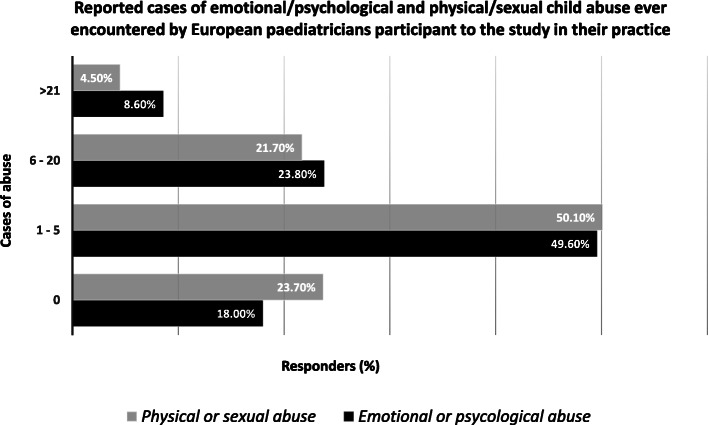
Reported cases of emotional/psychological and physical/sexual child abuse ever encountered by European paediatricians participant to the study in their practice. Percent of paediatricians who reported cases of physical/sexual and emotional/psychological child abuse, and number of cases ever observed in their practice

**Fig. 2:**
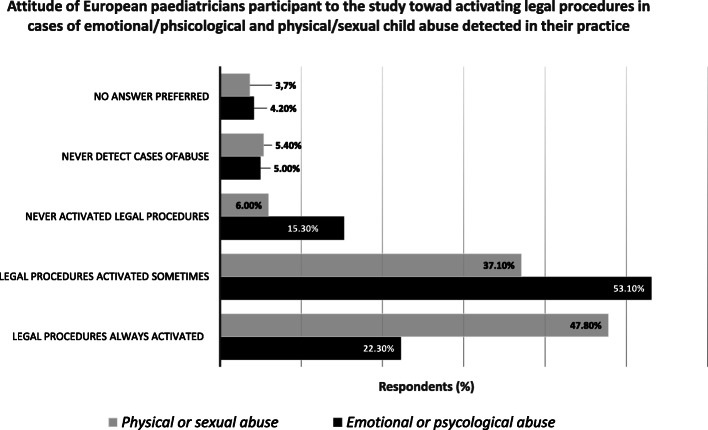
Attitude of European paediatricians participant to the study toward activating legal procedures in cases of emotional/psychological and physical/sexual child abuse detected in their practice. Percent of paediatricians who activated or did not activate legal procedures in presence of detected cases of emotional/psychological and physical/sexual child abuse in their practice

In the majority of cases in which CAN was identified by respondents, paediatricians were requested to visit children by one of the parents (41,7%) or by a different family member (21,6%). While the intervention of a paediatrician to recognize possible cases of child maltreatment was solicited by teachers or school officers in 7,9% of the cases and by family friends in 4,2%. In about one fourth of the cases (24,6%), the suspect of CAN was raised by other unspecified types of figures. Eighty per cent of respondents have encountered at least a case of emotional or psychological child abuse in their practice and 76.3% have faced at least a case of physical or sexual child abuse (Fig. 1). In the majority of these cases, paediatricians’ respondents have activated legal procedures in order to protect the victims (Fig. 2).

### Education and competence about CAN

One fourth (25,9%) of respondents rated their knowledge and competence about CAN to be good although improvable, and 2,6% to be excellent, while the majority of participants assessed their education on CAN as adequate (42,1%) and a minority not adequate (2,6%). During the three years preceding the study, about half of the respondents (47,8%) did not attend any continuing education course on CAN, versus 40,2% who attended educational programs addressing CAN, which in 39,2% of the cases were specialized courses held in person and 1.0% on line, while 12,0% attended generic in presence courses on domestic violence.

**Fig. 3:**
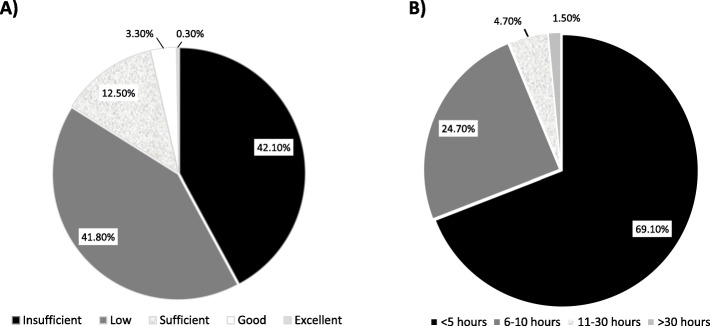
Knowledge about child abuse and neglect (CAN) provided in Europe by medical school curricula and paediatric residency programs, rated by European paedatricians participant to the study. A) Quality of educational programs on CAN provided in European medical schools and residency courses in paediatrics rated by paedatricians participant to the study (%). B) Reported number of training hours on CAN offered by European medical schools and paediatric residency courses, as reported by paediatricians participant to the study (%)

Knowledge about CAN provided in Europe by medical school curricula and paediatric residency programs was rated to be largely unsatisfactory, particularly due to the insufficient number of training hours dedicated to CAN (Fig. 3a and b). To this regard, the majority of paediatricians participants to the study indicated that educational programs on CAN should be made mandatory and included in the curricula of medical schools and residency courses in paediatrics, rating this option as useful (33.1) and necessary (65,5%), while only 1,4% consider that unnecessary.

### Practice and formal procedures about CAN

Local child protective services, including social services, were the first institutional point of reference in case of CAN for 64,1% of participants in the study, while for 22,4% of them local judicial authorities were their first choice to report a maltreatment. Contacting specialized hospital centers was the first option for 8,0% of respondents, 2,9% of them reported episodes of CAN to different institutions and 2,6% did not make any report.

83,6% of the paediatricians reported the existence of specific laws protecting victims of CAN in their countries, and two third (66,1%) confirmed the presence of standardized formal procedures that can be activated if cases of CAN are detected by doctors. However, 15,5% stated the absence of any formal procedure for the contrast of child maltreatment, while 18,5% of respondents were unaware of any form of procedure that could be activated in cases of CAN in their country. Although the large majority of respondents reported the existence of laws for the contrast of CAN in their countries (83,6%), only 52% rated these laws as adequate.

**Fig. 4:**
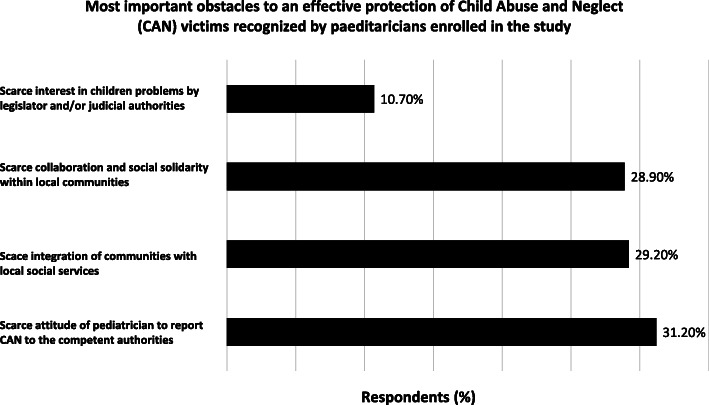
Most important obstacles to an effective protection of Child Abuse and Neglect (CAN) victims recognized by paediatricians enrolled in the study. List of major impediments to an effective management of CAN identified by paediatricians participant to the study (%)

The most important obstacles to an effective protection of CAN victims recognized by the paediatricians enrolled in the study are reported in Fig. 4. Finally, the large majority of respondents (88,0%) endorsed the statement that only a join action by child healthcare professionals at multi-national level, would provide an important lever to stimulate legislators to issue more effective laws and procedures to protect the victims of CAN.

## Discussion

Child maltreatment is a critical public health issue, with lifelong health consequences for victims and their families [26]. Detecting suspicious injuries may provide an important opportunity for early recognition and intervention to protect vulnerable children [2]. However, the identification ad report of suspected cases of CAN may be one of the most challenging and difficult tasks for pediatrician [26]. To identify initial revealing signs of abuse and manifestations of neglect requires professional competence acquired by adequate training, which cannot be improvised [27]. Maltreatment is usually recognized in clinical settings [26]. However, indicators of the various forms of child abuse are often nebulous and may be detected in different settings by various figures, including family friends, teachers and other members of the community related to the victims. Raising social awareness about maltreatment and close collaboration between members of local communities and paediatricians [28] are key factors to limit CAN and the phenomenon of domestic violence in general [29, 30]. During the past twenty years, a few previous studies have investigated attitudes and experiences of paediatricians on child maltreatment, however providing useful data regarding the progress and level of competence and awareness developed by paediatricians toward CAN worldwide [15, 31–33]. A recent study performed in Italy, about the competence of family paediatricians in Italy to identify child abuse, emphasized the scarce knowledge and ability of paediatricians and general practitioners to deal with child abuse and the importance of proper training programs [34]. However, studies on CAN performed in Europe are scarce, and the relatively small number of participants enrolled, somehow limited the importance of the information provided by the data analysis of these reports. The present study involved a number of participants significantly larger than previous studies. Furthermore, to our knowledge, no previous multi-national studies on attitudes and experiences of paediatricians on child maltreatment were performed in Europe, which could provide an updated overview at continental level.

Child abuse and neglect cases can be difficult to evaluate, and input from a trusted colleague, senior clinician, or medical specialists can be helpful [35]. The data of this study show a correlation between the number of reported cases of physical violence (> 5) and the age of paediatricians, as the higher the age of those enrolled in the questionnaire, the greater the number of findings they carried out, with a statistically significant difference (p 0.002; RR equal to 1.1).

Child maltreatment was recognized by the paediatricians participant to the study mostly during occasional visits (49,4%), and data analysis showed a significant correlation between detecting abuse during an occasional visit and being a primary care paediatrician (p 0.0034). In most of the European countries, if medical history or physical examination reveal suspicious signs and/or injuries, and in presence of a reasonable suspicion that a child has been abused, paediatricians are mandatorily required by law to report to child protective services or judicial authorities for further investigation. This study, showed a reluctance by paediatricians to report cases of CAN to the competent judicial authorities, while they mainly turn to child protection centres, which attitude correlated significantly with the reported finding of abuse (*p* > 0,05). There is a correlation between thinking that the laws in one’s country are adequate and having encountered physical and psychological abuse (p 0.002).

With proper education and training, most abuse can be prevented or interrupted [13]. Data of this study showed a statistically significant correlation between the number of training hours completed by respondents during their medical school and residency courses and those who believe they have sufficient education on CAN (p 0.002). The hours of academic training dedicated to this topic were also found to be statistically significant with respect to the cases of CAN identified, since paediatricians who recognized more than 5 cases had received a greater number of training hours on CAN (> 5), (p 0.2; RR 1.8) during their university studies. Cases of physical violence were detected in a greater percentage by paediatricians who had received a higher number of training hours during university studies (p 0.2). Furthermore, a large number of respondents reported to have acquired sufficient competence on CAN due to continuing education courses attended during the past three years (RR 1.8).

## Conclusions

Timely identification and intervention to protect violated children have the potential to stop the abuse, secure the child’s safety and prevent further stress in victims [36]. Paediatricians play an important role in the contrast of child maltreatment [37]. Data of this study particularly emphasize the importance to strengthen the knowledge on CAN through updating university curricula and specialist paediatric training, which for years have remained at levels that are currently not adequate to effectively contrast this phenomenon [38].

Our study was purposely limited to paediatricians and did not include family physicians, emergency physicians, advanced practice nurses or other medical personnel who care for children. In this regard, it captures the experience of those individuals considered most trained in the medical care and advocacy of children [32]. A further possible limitation of the study may be its cross-sectional design, based on analysing data of variables collected at one given point in time across a sample population or a pre-defined subset, and therefore any associations between educational and professional experiences and attitudes could not be considered causal. Limitations of survey studies due to various multiple factors cumulatively affecting their design, including number of questions and sample size are often unavoidable [39]. However, the data collected by this pilot study and their analysis may provide a useful contribution to the current limited knowledge about the familiarity of European paediatricians with child maltreatment and their skills to recognize, manage and contrast abusive childhood experiences in their practice. Finally, they could provide local legislators and health authorities with information useful to further improve public health approaches and rules able to effectively address shared risk and protective factors, which could prevent child abuse and neglect from ever occurring. Further multi-national studies focusing prevention and contrast of child abuse and neglect in the practice of European pediatricians would be useful to provide a better knowledge on CAN and its prevention.

## Data Availability

All data generated or analyzed during this study are included in this published article and are available from the corresponding author on reasonable request.

## Abbreviations

CAN: Child abuse and neglect
EPA/UNEPSA: European Paediatric Association/Union of National European Paediatric Societies and Associations
ECPCP: European Confederation of Primary Care Paediatricians
HKMA: Hong Kong Monetary Authority
